# Supplementary material for: A Critical View of the Application of the APEX Software (Aqueous Photochemistry of Environmentally-Occurring Xenobiotics) to Predict Photoreaction Kinetics in Surface Freshwaters
Source: Molecules. 2019 Dec 18;25(1):9. doi: 10.3390/molecules25010009 (PMC7017383; doi:10.3390/molecules25010009)
Supplement: Supplementary file 1 [file molecules-25-00009-s001.zip › 3.2.4_gcc-4.4.0.zip!/3.2.4_gcc-4.4.0/3.2.4_gcc-4.4.0/doc/octave/html/interpreter/Callbacks.html]

Callbacks - Untitled


Next: Object Groups,
Previous: Marker Styles,
Up: Advanced Plotting


---

#### 15.2.7 Callbacks

Callback functions can be associated with graphics objects and triggered
after certain events occur. The basic structure of all callback function
is

```
     function mycallback (src, data)
     ...
     endfunction
```

where `src` gives a handle to the source of the callback, and
`code` gives some event specific data. This can then be associated
with an object either at the objects creation or later with the
`set` function. For example

```
     plot (x, "DeleteFcn", @(s, e) disp("Window Deleted"))
```

where at the moment that the plot is deleted, the message "Window
Deleted" will be displayed.

Additional user arguments can be passed to callback functions, and will
be passed after the 2 default arguments. For example

```
     plot (x, "DeleteFcn", {@mycallback, "1"})
     ...
     function mycallback (src, data, a1)
       fprintf ("Closing plot %d\n", a1);
     endfunction
```

The basic callback functions that are available for all graphics objects
are

- CreateFcn
  This is the callback that is called at the moment of the objects
  creation. It is not called if the object is altered in any way, and so
  it only makes sense to define this callback in the function call that
  defines the object. Callbacks that are added to `CreateFcn` later with
  the `set` function will never be executed.- DeleteFcn
    This is the callback that is called at the moment an object is deleted.- ButtonDownFcn
      This is the callback that is called if a mouse button is pressed while
      the pointer is over this object. Note, that the gnuplot interface does
      not respect this callback.

The object and figure that the event occurred in that resulted in the
callback being called can be found with the `gcbo` and `gcbf`
functions.

— Function File: h = **gcbo** ()  
— Function File: [h, fig] = **gcbo** ()  
> Return a handle to the object whose callback is currently
> executing. If no callback is executing, this function returns the
> empty matrix. This handle is obtained from the root object property
> "CallbackObject".
>
> Additionally return the handle of the figure containing the
> object whose callback is currently executing. If no callback is
> executing, the second output is also set to the empty matrix.
>
>
>
> **See also:** gcf, gca, gcbf.

— Function File: fig = **gcbf** ()  
> Return a handle to the figure containing the object whose callback
> is currently executing. If no callback is executing, this function
> returns the empty matrix. The handle returned by this function is
> the same as the second output argument of gcbo.
>
>
>
> **See also:** gcf, gca, gcbo.

Callbacks can equally be added to properties with the `addlistener`
function described below.
